# *Notes from the Field:* Human Parvovirus B19 Infections Among Pregnant Persons — Minnesota, January–September 2024

**DOI:** 10.15585/mmwr.mm7347a4

**Published:** 2024-11-28

**Authors:** Stephen Contag, Elizabeth M. Dufort, Sarah Lim, Tyler Winkelman, Jennifer Zipprich, Lindsey Erickson, Mel Anacker, Nayanjot Kaur Rai, Kristen Ojo, Danielle Kvasager, Henry D. Kunerth, R. Adams Dudley, Pamala Gahr, Kelly R. Bergmann, Alanna M. Chamberlain, Summer Martins, Stephen Waring, Bjorn Westgard, Kristin Sweet, Paul Drawz, Ruth Lynfield

**Affiliations:** ^1^Division of Maternal-Fetal Medicine, University of Minnesota, Minneapolis, Minnesota; ^2^Minnesota Department of Health; ^3^Research and Evaluation Data Analytics Core, Hennepin Healthcare Research Institute, Minneapolis, Minnesota; ^4^Division of Nephrology and Hypertension, University of Minnesota, Minneapolis, Minnesota; ^5^University of Minnesota, Minneapolis Veterans Affairs Medical Center, Minneapolis, Minnesota; ^6^Children’s Minnesota, Minneapolis, Minnesota; ^7^Mayo Clinic, Rochester, Minnesota; ^8^Allina Health, Minneapolis, Minnesota; ^9^Essentia Institute of Rural Health, Duluth, Minnesota; ^10^HealthPartners Institute, Minneapolis, Minnesota.

**Summary**

**What is already known about this topic?**

Human parvovirus B19 (B19) infection during pregnancy can have serious consequences for the pregnancy and the fetus.

**What is added by this report?**

An increased frequency of B19 infections, including severe sequelae among pregnant women in Minnesota, was identified in 2024 through clinician reporting and evaluation of electronic health data.

**What are the implications for public health practice?**

Health care providers should have a high index of suspicion for B19 in pregnant persons, offer counseling, and provide appropriate monitoring and care.

Human parvovirus B19 (B19) commonly causes asymptomatic infection or mild illness in healthy children and nonpregnant adults, but infection during pregnancy can lead to severe perinatal sequelae, particularly when infection occurs before 20 weeks’ gestation. In July 2024, the Minnesota Department of Health (MDH) was notified by a maternal-fetal medicine specialist of an increase in B19 infections among pregnant persons associated with fetal complications. Although increased circulation of B19 had not been described in the United States at that time, review of the literature revealed that European surveillance indicated increases in B19 in late 2023 and 2024 identified through laboratory, clinical, and blood donation screening data (*1*,*2*).

## Investigation and Outcomes

Five cases of B19 infection among women aged 20–40 years who were evaluated during May–August 2024 were reported to MDH. No known epidemiologic links among the patients were identified, and the patients did not live in the same communities. Four patients had children in the household, including two who had ill children (one with B19-associated anemia requiring transfusion) and one who reported B19 circulating at her child’s school. The fifth patient had presumed exposure as a provider at a child care facility where febrile rash illnesses were circulating among attendees.

Three patients had signs and symptoms consistent with B19 infection, including fever, rash, malaise, fatigue, arthralgias, and lymphadenopathy. All five patients had B19 infection at 13–20 weeks’ gestation, laboratory-confirmed by immunoglobulin M or polymerase chain reaction (PCR) testing, including three who received positive B19 PCR amniotic fluid test results. None had immunocompromising conditions or blood disorders.

Patient A had fetal hydrops and experienced fetal demise at 20 weeks’ gestation before fetal transfusion could be performed. Patient B was evaluated weekly for 3 months and did not experience complications; patients C and D developed severe fetal anemia requiring fetal transfusion; and patient E developed severe fetal anemia with hydrops (severe edema) requiring two fetal transfusions. Patients B, C, D, and E delivered full-term infants with no birth or neonatal complications identified (Supplementary Table, https://stacks.cdc.gov/view/cdc/170371).

The MDH Public Health Laboratory performed metagenomic sequencing to generate full genomes using amniotic fluid from two patients. Both samples were genotype 1A, the most commonly circulating genotype worldwide. Specimens differed by at least 35 single nucleotide polymorphisms and did not appear to be related through a recent shared source or transmission event. Comparisons to sequences available in the National Center for Biotechnology Information (https://www.ncbi.nlm.nih.gov/) database revealed no closely related sequences based on single nucleotide polymorphisms or clustering on a phylogenetic tree.

No routine surveillance for B19 exists in the United States. It is not a notifiable condition, and it is not a reportable disease in Minnesota. To evaluate overall B19 trends and frequency of pregnancy-associated complications, the Midwest Analytics and Disease Modeling Center^§^ analyzed electronic health record data from 10 health systems provided through the Minnesota Electronic Health Record Consortium, among Minnesota residents during January 2019–September 2024 (Figure). This analysis identified an increase in parvovirus testing, positive tests, percentage of positive test results, and diagnoses in 2024 compared with 2019–2023, with the largest increases among children. During the 10-month period from January through September 2024, procedure and diagnosis codes identified 19 B19-associated pregnancy complications within 60 days of a B19 diagnosis or positive test result, including hydrops fetalis, fetal anemia and thrombocytopenia, fetal transfusion, or stillbirth. In comparison, during a 60-month period (2019–2023), 28 B19-associated pregnancy complications occurred. No increase in non–B19-associated fetal complications was identified during January–September 2024 compared with January 2019–December 2023.

**FIGURE:**
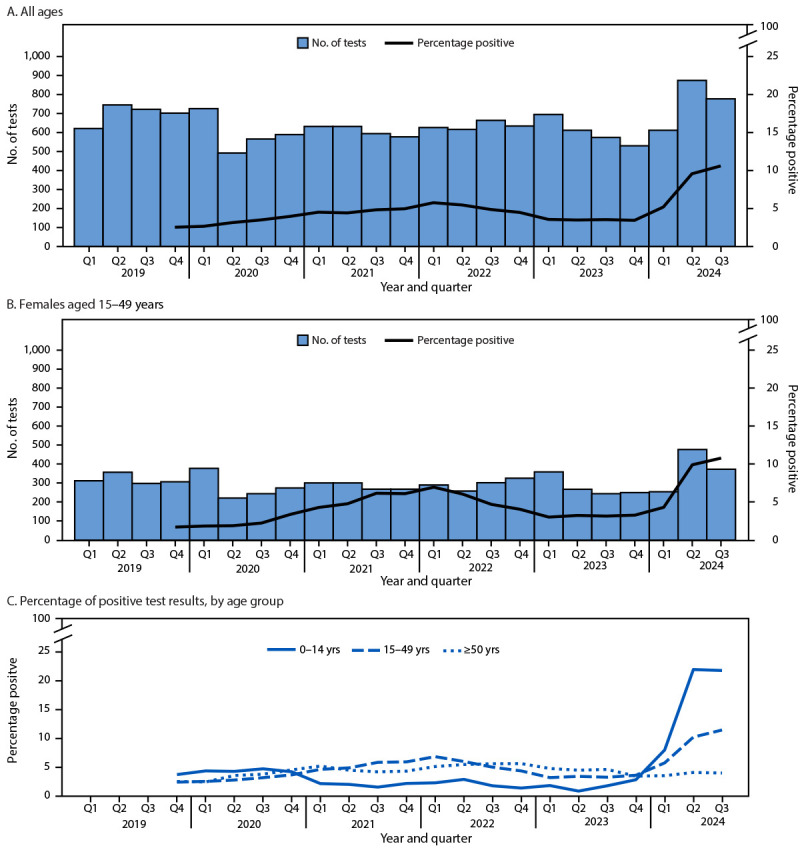
Number of human parvovirus B19 immunoglobulin M and polymerase chain reaction tests performed and percentage of tests with positive results* among persons of all ages (A), among females aged 15–49 years (B), and percentage of positive results by age group (C) — Minnesota, January 2019–September 2024^†^^,^^§^ **Abbreviation:** Q = quarter. * One-year moving average. ^†^ Q1 = January–March; Q2 = April–June; Q3 = July–September; Q4 = October–December. ^§^ A total of 10 health systems reported human parvovirus B19 test results through the Minnesota Electronic Health Record Consortium during January 2019–September 2024. Two health systems had data through June 2024. The remaining eight health systems had data through August 31, 2024; however, data from September 2024 is incomplete.

## Preliminary Conclusions and Actions

MDH alerted CDC and both agencies released health advisories (*3*,*4*). Health care providers should educate patients with suspected or confirmed B19 infection to inform exposed contacts who are pregnant and others at risk (such as those who are immunosuppressed or have chronic hemolytic blood disorders) and advise exposed contacts to consult with their health care providers. Obstetric providers should maintain a high index of suspicion for B19 and recommend testing (including serology and PCR) for pregnant persons with exposure to B19 or who have compatible signs and symptoms of maternal or fetal B19 disease, as clinically appropriate. Pregnant persons with B19 infection should be evaluated for fetal or pregnancy complications by an obstetric specialist (*5*). Public health officials should raise awareness about parvovirus B19 activity, including among child care and school providers, and provide information about who might be at higher risk for severe B19 disease and when infected children and staff members can return to child care or school after infection.

## Footnotes

* These authors contributed equally to this report.

† These senior authors contributed equally to this report.

§ Midwest Analytics and Disease Modeling Center is a CDC Center for Forecasting and Outbreak Analytics–funded partnership among the University of Minnesota, the Minnesota Department of Health, and the Minnesota Electronic Health Records Consortium (a collaboration among the 11 largest health systems that collectively care for >90% of Minnesotans). https://www.sph.umn.edu/research/centers/midwest-analytics-and-disease-modeling/

## References

[1] Russcher A, Verweij EJT, Maurice P, Extreme upsurge of parvovirus B19 resulting in severe fetal morbidity and mortality. Lancet Infect Dis 2024;24:e475–6. 10.1016/S1473-3099(24)00373-6 38901439

[2] European Centre for Disease Prevention and Control. Risks posed by reported increased circulation of human parvovirus B19 in the EU/EEA. Stockholm, Sweden: European Centre for Disease Control and Prevention; 2024. https://www.ecdc.europa.eu/en/publications-data/risks-posed-reported-increased-circulation-human-parvovirus-b19-eueea

[3] CDC. Increase in human parvovirus B19 activity in the United States. Health Alert Network (HAN). Atlanta, GA: US Department of Health and Human Services, CDC; 2024. https://emergency.cdc.gov/han/2024/han00514.asp

[4] Minnesota Department of Health. Health advisory: increase in human parvovirus B19. Minneapolis, MN: Minnesota Department of Health; 2024. https://www.health.state.mn.us/communities/ep/han/2024/aug16parvo.pdf

[5] American College of Obstetricians and Gynecologists. Practice bulletin no. 151: cytomegalovirus, parvovirus B19, varicella zoster, and toxoplasmosis in pregnancy. Obstet Gynecol 2015;125:1510–25. 10.1097/01.AOG.0000466430.19823.53 26000539

